# Radiologically Guided Renal Artery Embolization with an Amplatzer Vascular Plug as a Rescue Therapy for Refractory Nephrotic Syndrome in AL-Amyloidosis

**DOI:** 10.1155/2019/5469712

**Published:** 2019-02-11

**Authors:** I. Spozio Züst, H. R. Räz, F. Burkhalter

**Affiliations:** ^1^Division of Nephology, Kantonsspital Baden, Switzerland; ^2^Clinic for Transplant Immunology and Nephrology, University Hospital Basel, Switzerland

## Abstract

Nephrotic syndrome is common in immunoglobulin light-chain (AL) amyloidosis and successful therapy may pose a challenge. We report the case of a 63-year-old patient with severe nephrotic syndrome due to primary renal AL-amyloidosis with well-preserved renal function at first presentation. Therapy with high dose steroids, loop diuretics, and ACE-inhibitors did not affect his proteinuria and he was seriously disabled because of symptomatic orthostatic hypotension and anasarca. With the patient's informed consent, medical nephrectomy was tried with nonsteroidal-anti-inflammatory drugs (NSAIDs), cyclosporine, and aminoglycosides, with significant deterioration of his renal function, but without relevant effect on his proteinuria. Despite adequate anticoagulation life threatening thrombotic and bleeding complications occurred. Total renal ablation was finally achieved using an Amplatzer vascular plug Typ IV (AVP 4) with a self-expanding Nitinol mesh design, which was placed in both main renal arteries in the same intervention. The patient became completely anuric, protein loss stopped, and serum albumin slowly rose to normal levels. The patient's clinical condition dramatically improved and he regained his full mobility at the price of a lifelong renal replacement therapy. To our knowledge, this is the first reported usage of such a vascular occluder in the setting of refractory nephrotic syndrome with normal kidney function at the time of first presentation.

## 1. Background

Nephrotic syndrome refractory to conventional therapy is a well-known complication of renal light chain (AL) amyloidosis. Beside the treatment of the underlying disease, therapy options are symptomatic and include low-salt diet, diuretics, ACE-inhibitors, and angiotensin-receptor blockers (ARB). In unaffected severe proteinuria medical nephrectomy with NSAIDs, calcineurin-inhibitors, aminoglycosides or radio-contrast-agents may be tried. As a treatment of last resort, surgical nephrectomy needs to be discussed or alternatively radiologically guided embolization of the renal arteries can be an option. Bilateral renal artery embolization was developed in the early nineteen-seventies as a treatment for renal cell carcinoma [1, 2] and was first described 1975 for the treatment of nephrotic syndrome [3]. For renal artery embolization, various materials are used with different success- and complication rates [4, 5]. A common side effect is postembolization syndrome with a reported frequency of 47-100% [6]. It is characterized by fever, flank pain, leukocytosis, and an elevated lactate dehydrogenase in the first few hours up to a few days after the embolization procedure.

## 2. Case

We report the case of a 63-year-old patient who presented to the nephrology department with an overt nephrotic syndrome and a normal kidney function. He had a history of arterial hypertension under medical treatment for several years. Our investigations revealed the presence of a light chain plasma cell dyscrasia IgG kappa and IgA kappa/lambda with a Bence Jones proteinuria type lambda, not fulfilling the criteria of a multiple myeloma on bone marrow biopsy (plasma cell infiltration < 10%). A kidney biopsy revealed a severe diffuse glomerular, vascular, and interstitial amyloidosis of the AL-lambda type with slight interstitial fibrosis (10%) and tubular atrophy. Immediately after the kidney biopsy, a self-limiting macrohematuria occurred and at the same time an isolated muscular vein thrombosis of the left lower leg was diagnosed. The next day the patient was admitted to the emergency unit with a subacute ST-elevation myocardial infarction. Coronary angiography showed a primary spiral dissection with occlusion of the left anterior descending artery (LAD). In the echocardiography the left ventricular function appeared moderately reduced with anterior dyskinesia, not suggestive of a cardiac amyloidosis. In addition to aspirin, an oral anticoagulation with dicoumarol was started. Meanwhile, the patient's urinary protein/creatinine ratio rose to 2175 mg/mmol, the serum albumin level dropped to 11 g/l, and he developed anasarca. Severe orthostatic hypotension with several falls made him completely bed-ridden. Medical nephrectomy to reduce proteinuria was started with initially Celecoxib (100 mg bid) due to unchanged proteinuria additionally with cyclosporine (200mg bid). A further rise of creatinine was observed up to 400 *μ*mol/l, but urine volume and proteinuria did not decline, and the clinical condition of the patient worsened. To ameliorate overhydration and due to the reduced renal function, haemodialysis was started. The patient's condition improved slightly and his body weight could be stabilized but his urine volume remained between 0.5 and 1.5 litres per day. Still he remained severely orthostatic and was completely immobilized. Repeated echocardiography showed a severe reduced left ventricular function with a thrombus in the left ventricle despite oral anticoagulation and a pelvic deep venous thrombosis developed additionally. Dicoumarol and thalidomide were stopped and low molecular weight heparin (LMWH) was started. Two weeks later, the patient suffered an ischemic insult in the region of the posterior cerebral artery. LMWH was replaced by unfractionated heparin. He was in critical clinical condition and the decision for a renal ablation with radiologically guided embolization of both renal arteries was made in order to stop the proteinuria and save the patient's life. Two AVP 4 were placed by an interventional radiologist through a 5-F SOS catheter in both renal arteries using a standard femoral artery approach. The result was a complete occlusion of the left renal artery and an almost complete occlusion of the right renal artery. During the procedure, the patient received an epidural anaesthesia with bupivacaine and a prophylactic antibiotic treatment with piperacillin/tazobactam. Despite his severe illness, the patient tolerated the intervention well. A moderate postembolization syndrome developed with ischemic flank pain and fever up to 38.1°C, and LDH rose up to 786 U/l. The pain could be controlled by continuing the epidural anaesthesia for 48 h. Urinary output ceased completely, serum albumin levels normalized slowly during follow-up, and prophylactic antibiotic treatment was stopped after 5 days and normalization of the transient elevated C-reactive protein. The patient's general condition improved dramatically. He could be fully mobilized again, and over time he was able to go regularly for a walk and take a few laps in his swimming pool daily with an acceptable quality of life on maintenance hemodialysis. Without any further chemotherapy however, signs of systemic amyloidosis progressively developed 4 years after the intervention. Over time liver and spleen massively enlarged, ascites developed and recurrent pleural effusion was successfully treated by Talcum-Pleurodesis. As consequence of coronary heart disease and chronic renocardial syndrome, left ventricular ejection fracture worsened again. The patient complained of fading strength of his legs with difficulty to climb stairs and finally 8 years after the intervention he died from the consequences of systemic amyloidosis.

## 3. Discussion

Our patient suffered from life threatening nephrotic syndrome caused by renal amyloidosis of the AL-Lambda Type due to a plasma cell dyscrasia not fulfilling the diagnostic criteria of a multiple myeloma. Because of severe hypoalbuminemia, generalized oedema, and immobilizing orthostasis an unsuccessful medical nephrectomy was performed. After further several thrombotic and bleeding complications despite adequate anticoagulation, nephrectomy was considered as last resort. Regarding the desperate clinical condition of the patient, a surgical nephrectomy was considered inacceptable. To induce anuria, we consequently decided to embolize both renal arteries. This technique was developed in the early nineteen-seventies as a palliative therapy for surgically unresectable renal cell carcinoma. Soon after the first successful reports in cancer patients, the use of this technique was expanded to other indications (Table 1).

**Figure 1:**
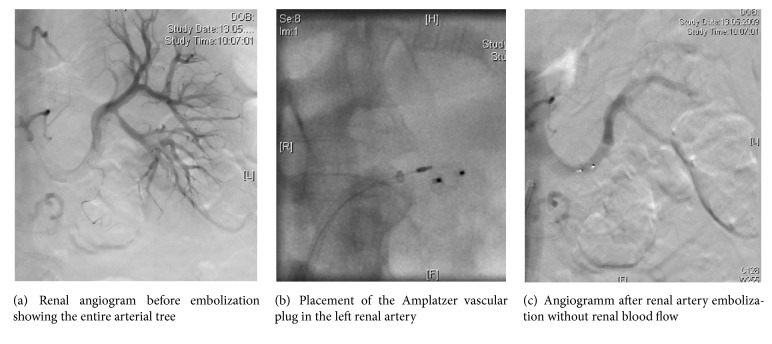


Data of total renal embolization are sparse and anecdotic and restricted to a few case reports and small case series [4, 7–13]. Controlled data are missing only reflecting the fact that such a radical procedure, with the consequence of lifetime dialysis, remains a rescue therapy after all other options have failed [10–13]. The technique is not standardized. There are descriptions of a great variety of materials used as emboli, depending on the desired arterial occlusion-level, i.e., the level of arteries at which occlusion should take place (main artery, segmental or subsegmental arteries, and capillaries). The complications of the technique depend on the embolization-material used. As a rule of thumb complication rate rises with the use of smaller particles occluding at a lower vascular level. Ethanol, cyanoacrylate (“superglue"), and maize starch occlude at the capillary level. Segmental blocks are achieved with gelatine or acrylate spheres. To occlude the main renal artery directly, balloons, metal coils, and self-expanding wire meshes are used. The most common side effect of the procedure is postembolization syndrome with a reported frequency of 47-100% [6]. In the literature, the syndrome is characterized by fever, bilateral flank pain, leukocytosis, elevated blood sedimentation rate, rising levels of C-reactive protein, and lactate dehydrogenase. Subileus may occur. It is generally a benign condition of a few days duration and responds well to symptomatic treatment with nonsteroidal anti-inflammatory drugs or steroids. It is considered to be the consequence of kidney tissue necrosis. Sepsis and abscess formation due to super infection of a necrotic kidney have rarely been described [14]. In nephrotic syndrome technical success rates with renal embolization of 100% are reported [7, 8, 10–13, 15]. With embolization on the capillary level, anuria normally develops. Accidental embolization of the infrarenal vascular bed has been reported to occur in around ten percent of patients [6], especially when small embolization particles or fluids are used. This can be prevented by transient proximal occlusion of the main renal artery with a balloon prior to injection of the embolization material. For our patient we chose the technique that was available, easily feasible, and allowing the occlusion of both renal arteries in the same session. We used an AVP 4, a self-expanding wire mesh (Nitinol®), which is normally used for peripheral vascular embolization [16]. It is placed under radiological guidance by coaxial technique (Figure 1). The advantage of using an AVP 4 is that it can be delivered through a 0.038 inch guidewire-compatible diagnostic catheter and so eliminates the need for sheath or guiding catheter exchange. In contrast to coils, there is a higher success rate of complete occlusion, a low risk of distal embolization, and the possibility of repositioning if needed. In order to prevent ischemic pain, the patient received epidural anesthesia. After this proximal embolization with an Amplatzer device the patient continued to produce very low amounts of urine due to residual blood supply from renal capsular vessels. Postembolization syndrome was only mild with fever up to 38.1°C and a slightly elevated LDH. After the procedure, our patient recovered, was able to walk 4 km daily, and had the chance to live further 8 years with an acceptable quality of life.

In conclusion, proximal embolization of both kidneys with Amplatzer vascular plugs in a single intervention is feasible, safe, and successful and gave our patient the chance to continue his life with an acceptable quality of life for several years at the price of lifelong dialysis dependency. The percutaneous embolization of the kidneys is a valid and alternative procedure to the surgery with a lower risk for complications and high success rate using an AVP 4.

## Figures and Tables

**Table 1:** Reported indications of renal embolization.

Palliative treatment of renal cell carcinoma

Refractory renal hypertension in ESRD or transplant patients

Transplant rejection

Nephrotic syndrome refractory to treatment

Chronic urinary fistulas

Traumatic renal hemorrhage
